# Supplementary material for: Design and optimisation of dendrimer-conjugated Bcl-2/xL inhibitor, AZD0466, with improved therapeutic index for cancer therapy
Source: Commun Biol. 2021 Jan 25;4:112. doi: 10.1038/s42003-020-01631-8 (PMC7835349; doi:10.1038/s42003-020-01631-8)
Supplement: Supplementary file 2 — Supplementary Information [file 42003_2020_1631_MOESM2_ESM.pdf]

Supplementary Information for

Design and optimisation of dendrimer-conjugated Bcl-2/x_L inhibitor, AZD0466, with improved therapeutic index for cancer therapy

Authors: Claire M. Patterson[1$], Srividya B. Balachander[2$], Iain Grant[3$], Petar Pop-Damkov[2], Brian Kelly[7], William McCoull[6], Jeremy Parker[1], Michael Giannis[7], Kathryn J. Hill[3], Francis D. Gibbons[2], Edward J. Hennessy[2], Paul Kemmitt[6], Alexander R. Harmer[4], Sonya Gales[4], Stuart Purbrick[4], Sean Redmond[5], Matthew Skinner [4], Lorraine Graham[1], J. Paul Secrist[2], Alwin G. Schuller[2], Shenghua Wen[2], Ammar Adam[2], Corinne Reimer [2], Justin Cidado[2], Martin Wild[6], Eric Gangl[2], Stephen E. Fawell [2], Jamal Saeh[2], Barry R. Davies[6] , David J. Owen[7] Marianne B. Ashford[1]*

Affiliations:

[1] Pharmaceutical Sciences, R&D, AstraZeneca, Macclesfield, UK.

[2]Oncology R&D, AstraZeneca, Boston, USA.

[3]Pharmaceutical Technology and Development, Operations, AstraZeneca, Macclesfield, UK.

[4]Clinical Pharmacology & Safety Sciences, R&D, AstraZeneca, Cambridge, UK.

[5]Clinical Pharmacology & Safety Sciences, R&D, AstraZeneca, Boston, USA.

[6]Oncology R&D, AstraZeneca, Cambridge, UK.

[7]Starpharma, 4-6 Southampton Crescent, Abbotsford, Victoria, 3067, Australia.

*To whom correspondence should be addressed: E-mail: Marianne.Ashford@astrazeneca.com

[$]These authors contributed equally

Supplementary Information

Supplementary Methods

Preparation of Glutarate Linked AZD4230 Dendrimer Conjugates (SPL-8931)

To a magnetically stirred suspension of AZD4320 (200 mg, 0.21 mmol) in DCM (10 ml) at room temperature glutaric anhydride (29 mg, 0.25 mmol), DMAP (26 mg, 0.21 mmol) and DIPEA (93 μL, 0.53 mmol) were added. The suspension dissolved quickly, and the mixture was left to stir at room temperature overnight. Additional glutaric anhydride was added over the following 24

hours until the reaction was judged >80% complete by HPLC. The volatiles were then removed in vacuo and the residue purified by preparative HPLC (BEH 300 Waters XBridge C18, 5 µM, 30 x 150 mm, 60-80% ACN/water (5-40 min), 0.1% TFA, RT = 22 min) providing 117 mg (52%) of the AZD4320 Glutarate as a white solid.

To a magnetically stirred mixture of AZD4320 Glutarate (67 mg, 63 µmol) and PyBOP (33 mg, 63.3 µmol) in DMF (1 ml) at room temperature, a mixture of G5 PEGylated dendrimer (Fig. S3. [Y = PEG2100, Q = H.TFA salt], 99 mg, 1.32 µmol) and NMM (23 µL, 0.21 mmol), also in DMF (2 ml) was added. After 16 hours at room temperature the volatiles were removed, and the residue purified by size exclusion chromatography (sephadex, LH20, MeOH). The appropriate fractions, as judged by HPLC, were combined and concentrated. The residue was then taken up in water, filtered (0.22 µm) and lyophilised, providing 101 mg (73%, Fig. S4.) of desired material as a pale pink solid. HPLC (C8 Xbridge, 3 x 100 mm, gradient: 42-50% ACN/H2O) (1-7 min), 50-80% ACN (7-8 min), 80% ACN (8-11 min), 80-42% ACN (11-12 min), 42% ACN (12-15 min), 214 nm, 10 mM ammonium formate) Rf (min) = 10.18. 1H-NMR (300MHz, CD3OD) δ (ppm): 0.65-2.08 (m, 585H), 2.10-2.50 (m, 144H), 2.50-2.80 (m, 71H), 2.82-3.02 (m, 80H), 3.04-3.27 (m, 137H), 3.35 (s, 108H), 3.40-4.06 (m, 5824H), 4.08-4.62 (m, 181H), 6.54-8.40 (m, 632H).

Preparation of Thiodiglycolate Linked AZD4230 Dendrimer Conjugate (SPL-8932)

To a magnetically stirred suspension of AZD4320 (70 mg, 74.1 µmol) in DCM (5 ml) at room temperature thiodiglycolic anhydride (TDA, 10 mg, 74.1 µmol) and DIPEA (33 µl, 185 µmol) were added. The suspension dissolved quickly, and the mixture was left to stir at room

temperature overnight. Additional thiodiglycolic anhydride was added over the following 24 hours until the reaction was judged >80% complete by HPLC. The volatiles were then removed in vacuo and the residue purified by preparative HPLC (BEH 300 Waters XBridge C18, 5 µM, 30 x 150 mm, 60-80% ACN/water (5-40 min), 0.1% TFA, RT = 22 min) providing 63 mg (70%) of AZD4320 thiodiglycolate as a white solid.

To a magnetically stirred mixture of AZD4320 thiodiglycolate (62 mg, 58 µmol) and PyBOP (30 mg, 58 µmol) in DMF (1 ml) at room temperature, a mixture of G5 PEGylated dendrimer (Fig. S3 [Y = PEG2100, Q = H.TFA salt], 97 mg, 1.28 µmol) and NMM (27 µl, 0.24 mmol), also in DMF (2 ml) was added. After 16 hours at room temperature the volatiles were removed, and the residue purified by size exclusion chromatography (sephadex, LH20, MeOH). The appropriate fractions, as judged by HPLC, were combined and concentrated. The residue was then taken up in water, filtered (0.22 µm) and lyophilised, providing 98 mg (72%, Fig. S5.) of desired material as a pale pink solid. HPLC (C8 Xbridge, 3 x 100 mm, gradient: 42-50% ACN/H2O) (1-7 min), 50-80% ACN (7-8 min), 80% ACN (8-11 min), 80-42% ACN (11-12 min), 42% ACN (12-15 min), 214 nm, 10 mM ammonium formate) Rf (min) = 10.24. 1H-NMR (300MHz, CD3OD) δ (ppm): 0.62-2.33 (m, 589H), 2.37-2.69 (m, 87H), 2.69-2.92 (m, 98H), 2.94-3.27 (m, 202H), 3.35 (s, 113H), 3.37-4.10 (m, 5781H), 4.10-4.70 (m, 154H), 6.50-8.45 (m, 661H).

Preparation of Diglycolate Linked AZD4230 Dendrimer Conjugate (SPL-8933)

To a magnetically stirred suspension of AZD4320 (77 mg, 81.5 µmol) in DCM (5 ml) at room temperature, diglycolic anhydride (9.6 mg, 81.5 µmol) and DIPEA (36 µL, 200 µmol) were added. The suspension dissolved quickly and the mixture was left to stir at room temperature

overnight. Additional diglycolic anhydride was added over the following 24 hours until the reaction was judged >80% complete by HPLC. The volatiles were then removed in vacuo and the residue purified by preparative HPLC (BEH 300 Waters XBridge C18, 5 µM, 30 x 150 mm, 60-80% ACN/water (5-40 min), 0.1% TFA, RT = 22 min) providing 76 mg (87%) of AZD4320 diglycolate as a white solid.

To a magnetically stirred mixture of AZD4320 diglycolate (76 mg, 72 µmol) and PyBOP (37 mg, 72 µmol) in DMF (1 ml) at room temperature, a mixture of G5 PEGylated dendrimer (Fig. S3 [Y = PEG2100, Q = H.TFA salt], 112 mg, 1.49 µmol,) and NMM (31 µl, 0.29 mmol), also in DMF (2 ml) was added. After 16 hours at room temperature the volatiles were removed and the residue purified by size exclusion chromatography (sephadex, LH20, MeOH). The appropriate fractions, as judged by HPLC, were combined and concentrated. The residue was then taken up in water, filtered (0.22 µm) and lyophilised, providing 137 mg (88%, Fig. S6.) of desired material as a pale pink solid. HPLC (C8 Xbridge, 3 x 100 mm, gradient: 42-50% ACN/H2O) (1-7 min), 50-80% ACN (7-8 min), 80% ACN (8-11 min), 80-42% ACN (11-12 min), 42% ACN (12-15 min), 214 nm, 10 mM ammonium formate) Rf (min) = 10.23. 1H-NMR (300MHz, CD3OD) δ (ppm): 0.58-2.26 (m, 834H), 2.28-2.72 (m, 154H), 2.74-3.28 (m, 245H), 3.35 (s, 101H), 3.37-4.02 (m, 5824H), 4.04-4.68 (m, 272H), 6.46-8.54 (m, 652H).

Preparation of N-Methyl Diacetate Linked AZD4230 Dendrimer Conjugate (SPL-8977)

To a magnetically stirred suspension of AZD4320 (200 mg, 0.21 mmol) in DCM (5 ml) at room temperature, a mixture of DIPEA (24 µl, 0.14 mmol), NMM (72 µL, 0.66 mmol) and 4-methylmorpholine-2,6-dione (33 mg, 0.26 mmol) were added. The suspension dissolved quickly

and the mixture was left to stir at room temperature overnight. Additional 4-methylmorpholine-2,6-dione was added over the following 24 hours until the reaction was judged >80% complete by HPLC. The volatiles were then removed in vacuo and the residue purified by preparative HPLC (BEH 300 Waters XBridge C18, 5 μM, 30 x 150 mm, 50-70% ACN/water (5-40 min), 0.1% TFA, RT = 23 min) providing 190 mg (84%) of AZD4320 N-Methyl Diacetate as a white solid.

To a magnetically stirred mixture of AZD4320 N-Methyl Diacetate (730 mg, 0.68 mmol) and PyBOP (353 mg, 0.68 mmol) in DMF (10 ml) at room temperature, a mixture of G5 PEGylated dendrimer (Fig. S3 [Y = PEG2100, Q = H.TFA salt], 934 mg, 12.1 μmol) and NMM (255 μl, 2.32 mmol), also in DMF (10 ml) was added. After 16 hours at room temperature the volatiles were removed, and the residue purified by size exclusion chromatography (sephadex, LH20, ACN). The appropriate fractions, as judged by HPLC, were combined and concentrated. The residue was then taken up in water, filtered (0.22 μm) and lyophilised, providing 1.19 g (92%, Fig. S12) of desired material as a pale pink solid. HPLC (C8 Xbridge, 3 x 100 mm, gradient: 42-50% ACN/H2O) (1-7 min), 50-80% ACN (7-8 min), 80% ACN (8-11 min), 80-42% ACN (11-12 min), 42% ACN (12-15 min), 214 nm, 10 mM ammonium formate) Rf (min) = 10.80. 1H-NMR (300MHz, CD3OD) δ (ppm): 0.45-1.92 (m, 565H), 2.08-2.78 (m, 228H), 2.79-3.00 (m, 96H), 3.01-3.28 (m, 180H), 3.35 (s, 180H), 3.46-4.20 (m, 6164H), 4.20-4.68 (m, 139H), 6.40-8.52 (m, 680H).

Particle size

The particle size distribution was determined by dynamic light scattering (DLS) of a dilute suspension in methanol at 25°C on a Brookhaven ZetaPALS instrument with a 660 nm laser

scattered at 90°. DLS data were analysed using the cumulants method. The particle size analysis was performed in triplicate and the average value for mean particle diameter is reported.

AZD4320 Load

AZD4320 loading was quantified by ^{1}H NMR spectroscopy.

In vitro release

AZD4320 release kinetics were determined in vitro under physiological conditions. Nanoparticles were suspended in phosphate buffered saline (PBS) pH7.4 or rat plasma with 5% HEPES in HPLC vials and sample temperature maintained to 37 °C using a water bath with continuous stirring of the sample provided by iChem explorer unit. Periodically, 5-10 µL of sample was injected onto the column (Acquity UPLC BEH shield RP18 1.7 µm, 4.6 x 30 mm, Waters), bracketed by DMA to re-dissolve any precipitated AZD4320. The percent release was calculated by comparing unconjugated AZD4320 peak area to total unconjugated + dendrimer-conjugated AZD4320 peak areas. The half-life was calculated by least squares fitting to a 1st order release model.

Derivation of closed form expressions for the mathematical model

The simplifying assumptions used in the modelling work allow the derivation of closed-form equations for the amounts and concentrations of released and dendrimer-conjugated AZD4320 in the tumour tissue. We also consider the dendrimer conjugated AZD4320 in systemic circulation (in plasma).

AZD4320 conjugated dendrimer in plasma

The rate of change of mass or amount of dendrimer conjugated active moiety in the plasma with respect to time (as the result of release of active in the plasma and uptake by the RES system), is described by the following differential equation,

$$\frac{\mathrm{d}X_{\mathrm{c},pl}}{\mathrm{d}t} = -\left(k_{res} + k_h\right) X_{c,pl} \qquad \text{- (S1)}$$

$X_{\mathrm{c},pl}$ - Mass of dendrimer conjugated active in plasma

k_{res} - A first order constant for RES uptake

k_h - A first order constant for active release by hydrolysis

Equation (S1) can be solved, in this case for a bolus dose, by direct integration, with the conditions that at time (t = 0), the initial mass of dendrimer conjugated drug in plasma is equivalent to the dose, to give,

$$X_{c,pl} = De^{-(k_{res}+k_h)t} \qquad \text{- (S2)}$$

D - Total dose of conjugated drug (as active equivalent)

Alternatively, in terms of concentration, we can write,

$$C_{c,pl} = \frac{D}{V_{pl}} e^{-(k_{res}+k_h)t} \qquad \text{- (S3)}$$

V_{pl} - The volume of plasma.

AZD4320 conjugated dendrimer in Tumour

For the dendrimer conjugated active moiety in tumour, the rate of change with respect to time is given by, the input rate due to extravasation from the plasma minus the removal rate due to hydrolysis of the conjugated active to released active.

$$\frac{\mathrm{d}X_{\mathrm{c},tu}}{\mathrm{d}t} = k_{ext} X_{\mathrm{c},pl} - k_h X_{\mathrm{c},tu} \qquad \text{- (S4)}$$

$X_{c,pl}$ - Mass of dendrimer conjugated active in tumour

k_{ext} - A first- order constant for extravasation of the dendrimer from plasma to tumour

We can substitute (S2) into (S4) to give (S5),

$$\frac{\mathrm{d}X_{\mathrm{c},tu}}{\mathrm{d}t} + k_h X_{\mathrm{c},tu} = k_{ext} D e^{-(k_{res}+k_h)t} \qquad \text{- (S5)}$$

Equation (S5) can be solved, for example using the integrating factor method (with $X_{c,tu} = 0$ at $t = 0$), to give equation (S6) for dendrimer conjugated active in the tumour tissue,

$$X_{c,tu} = D \frac{k_{ext}}{k_{res}} e^{-k_h t} \left(1 - e^{-k_{res} t}\right) \qquad \text{- (S6)}$$

As in the previous case, we can write this in terms of concentration as,

$$C_{c,tu} = \frac{D}{V_{tu}} \frac{k_{ext}}{k_{res}} e^{-k_h t} \left(1 - e^{-k_{res} t}\right) \qquad \text{- (S7)}$$

V_{tu} - The volume of the tumour.

Concentrations of released AZD4320 in tumour

For the released active in tumour, the rate of change with respect to time is given by the input rate due to hydrolysis of the conjugated active already present in the tumour minus the removal rate due to leakage out of the tumour back into systemic circulation.

$$\frac{\mathrm{d}X_{\mathrm{r},tu}}{\mathrm{d}t} = k_h X_{\mathrm{c},tu} - k_{tu} X_{\mathrm{r},tu} \qquad \text{- (S8)}$$

k_{tu} - A first order constant for leakage of released active back to systemic circulation.

If we substitute (S6) into (S8) we get,

$$\frac{dX_{r,tu}}{\mathrm{d}t}+k_{tu}X_{r,tu}=k_h D\frac{k_{ext}}{k_{res}}e^{-k_h t}\left(1-e^{-k_{res}t}\right) \qquad \text{- (S9)}$$

Equation (S9) is solved again using the integrating factor method (with $X_{r,tu}$ = 0 at t = 0) to give equation (S10) for released active in the tumour tissue,

$$X_{r,tu}(t)=\frac{Dk_{ext}k_h}{k_{res}}\left(\frac{k_{res}e^{-k_{tu}t}}{\left(k_h-k_{tu}+k_{res}\right)\left(k_h-k_{tu}\right)}+\frac{e^{-\left(k_h+k_{res}\right)t}}{\left(k_h-k_{tu}+k_{res}\right)}-\frac{e^{-k_h t}}{\left(k_h-k_{tu}\right)}\right) \qquad \text{- (S10)}$$

Finally, we can obtain the expression for the concentration of released drug at the tumour site by dividing through by the tumour volume to give,

$$C_{r,tu}(t)=\frac{Dk_{ext}k_h}{V_{tu}k_{res}}\left(\frac{k_{res}e^{-k_{tu}t}}{\left(k_h-k_{tu}+k_{res}\right)\left(k_h-k_{tu}\right)}+\frac{e^{-\left(k_h+k_{res}\right)t}}{\left(k_h-k_{tu}+k_{res}\right)}-\frac{e^{-k_h t}}{\left(k_h-k_{tu}\right)}\right) \qquad \text{- (S11)}$$

From equation (S11), it can be noted that k_{ext}, the extravasation constant, only occurs once in the above expression and acts simply as an overall scaling factor.

To find the maximum value of this function, equation (S12), the time derivative, should be solved for t using a numerical method, to get the time at which the maximum value occurs, t_{max}.

$$\frac{Dk_{ext}k_h}{V_{tu}k_{res}}\left(\frac{k_h e^{-k_h t}}{\left(k_h-k_{tu}\right)}-\frac{k_{tu}k_{res}e^{-k_{tu}t}}{\left(k_h-k_{tu}+k_{res}\right)\left(k_h-k_{tu}\right)}-\frac{\left(k_h+k_{res}\right)e^{-\left(k_h+k_{res}\right)t}}{\left(k_h-k_{tu}+k_{res}\right)}\right)=0 \qquad \text{- (S12)}$$

Using this solution for $t = t_{max}$ in equation (11) provides a value for the maximum possible concentration of released active in the tumour, for the given values of dose and hydrolysis half-life.

Prediction of released concentration of AZD4320 in plasma

A conventional 3-compartment pharmacokinetic (PK) modelling approach is used to predict the released concentration of the active moiety as resulting from bolus dosing of the dendrimer conjugates.

The 3-compartment model is parameterised using PK data from a conventional IV dose of the active moiety in a solution formulation.

We can write the follow differential equations describing the derivative of the amount of released active with respect to time.

For dendrimer conjugated active in plasma.

$$\frac{dX_0}{dt} = -\left(k_h + k_{res}\right) X_0 \qquad \text{- (S13)}$$

For the PK for plasma, Compartment 1

$$\frac{dX_1}{dt} = k_h X_0 - \left(k_{el} + k_{12} + k_{13}\right) X_1 + k_{21} X_2 + k_{31} X_3 \qquad \text{- (S14)}$$

Where $k_{el} = CL/V_c$, - CL denotes the plasma clearance and V_c, the central volume (of compartment 1)

PK for Compartment 2

$$\frac{dX_2}{dt} = k_{12}X_1 - k_{21}X_2 \qquad \text{- (S15)}$$

PK for Compartment 3

$$\frac{dX_3}{dt} = k_{13}X_1 - k_{31}X_3 \qquad \text{- (S16)}$$

Equations (S13 to S16) are collectively solved numerically, for example, using scipy.integrate.odeint (from SciPy 1.3.1, SciPy.org and Python 3.7.4, Python Software Foundation) with the initial conditions that $X_0 = D$ at $t = 0$ with the amounts in compartments 1 to 3 set to zero at $t = 0$. The predicted plasma concentration of release active is determined from the solution to equation (S14) divided by the central volume (V_c).

To obtain a closed form approximation for the release concentration of AZD4320 in plasma over a short time period, up to and including the plasma C_{max}, we can approximate equation (S14) as,

$$\frac{dX_1}{dt} \approx k_h X_0 - \left(k_{el} + k_{12} + k_{13}\right) X_1 \qquad \text{(S17)}$$

because the terms in X2 and X3 will be relatively small, until a reasonable time after dosing.

$$\frac{dX_1}{dt} + \left(k_{el} + k_{12} + k_{13}\right) X_1 = k_h D e^{-\left(k_{res} + k_h\right)t} \qquad \text{(S18)}$$

We solve equation (S18) with the condition that $X_1 = 0$ at $t = 0$, to give,

$$X_1 = \frac{D}{V_c}\left(\frac{k_h}{k_h + k_{res} - \left(k_{el} + k_{12} + k_{13}\right)}\right)\left(e^{-\left(k_{el} + k_{12} + k_{13}\right)t} - e^{-\left(k_h + k_{res}\right)t}\right) \qquad \text{(S19)}$$

Dividing the above by the central volume, gives the final approximate expression for the released concentration of AZD4320 in plasma.

$$C_{r,pl} = \frac{D}{V_c}\left(\frac{k_h}{k_h + k_{res} - \left(k_{el} + k_{12} + k_{13}\right)}\right)\left(e^{-\left(k_{el}+k_{12}+k_{13}\right)t} - e^{-\left(k_h+k_{res}\right)t}\right) \quad \text{(S20)}$$

Supplementary Figures

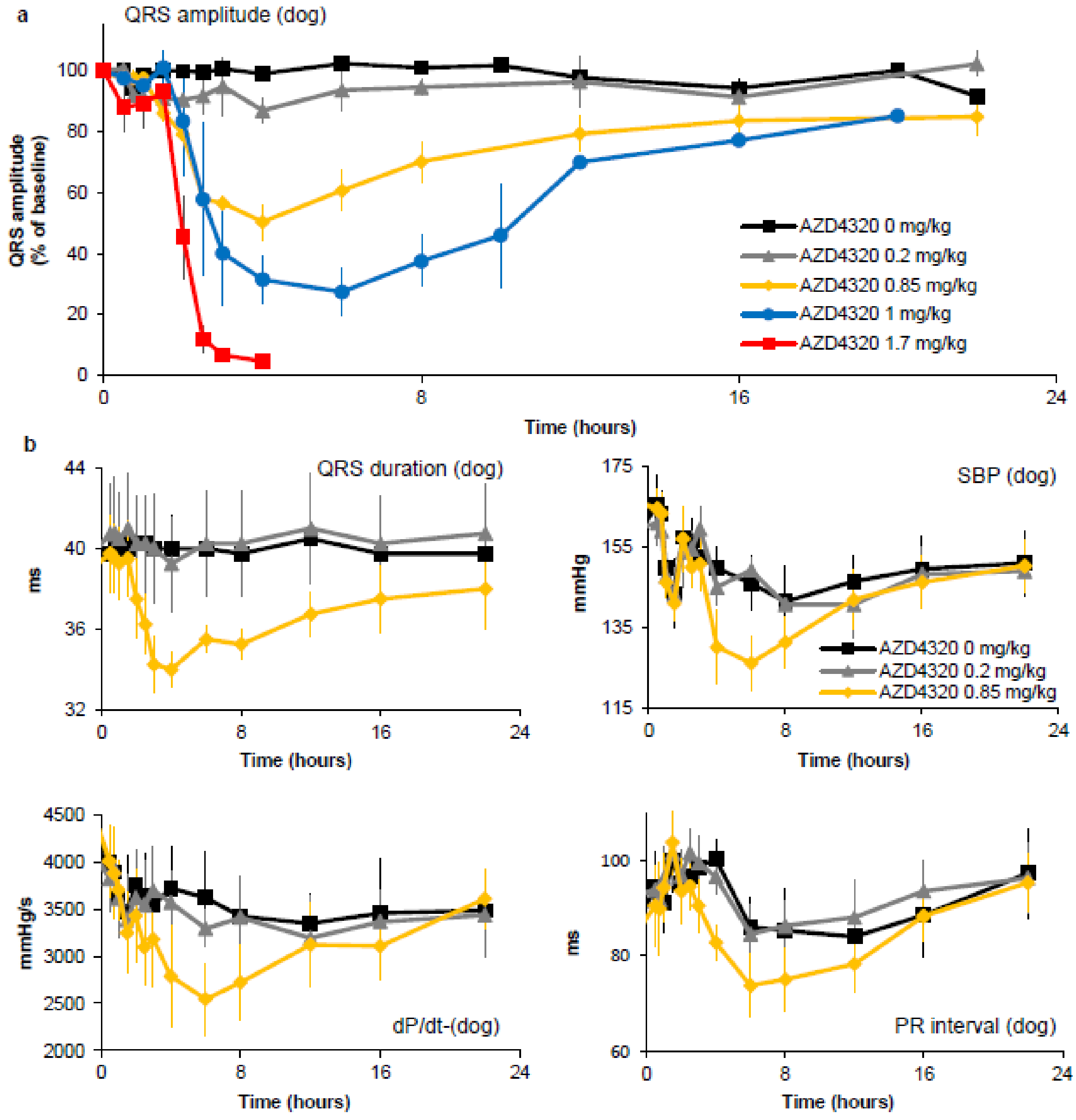

Supplementary Figure 1. Cardiovascular effects of AZD4320 in dog. Time course of cardiovascular effects in telemetered dogs following single dose IV administration at t = 0 of vehicle (0 mg/kg) or AZD4320 at different dose levels. Dose-response effects on QRS amplitude (a) and QRS duration, systolic blood pressure, dP/dt- and PR interval (b). Data are group mean ± SEM, n = 2 to 4 per group._0 mg/kg (black square) , 0.2 mg/kg–(grey triangles),0.85 mg/kg(orange diamonds), 1 mg/kg (blue circles), 1.7 mg/kg (red squares)

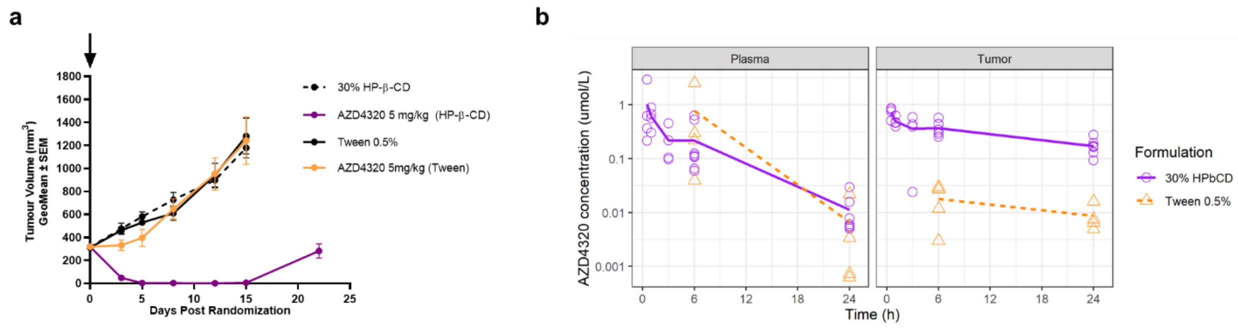

Supplementary Figure 2. Formulation dependent efficacy of AZD4320 (a) A single 5mg/kg IV bolus dose of AZD4320 formulated in 30% hydroxypropyl-β-cyclodextrin (HP-β-CD) (purple) causes complete regression of the tumours, while the same dose formulated in 0.5% Tween 80 (yellow) shows no efficacy. Data in the graphs are represented as mean tumor volume ± SEM (n=5). Arrow indicates dosing schedule.**(b)** A pharmacokinetic profile from both formulations shows that the lack of efficacy with 0.5% Tween 80 formulation is not simply explained by differential profiles in the plasma. The plasma profiles are similar. The efficacy is consistent with the concentration profiles observed in the tumour (n=4, Tween formulation, yellow, n=8 HP-β-CD formulation, purple). Lines connect the mean values.

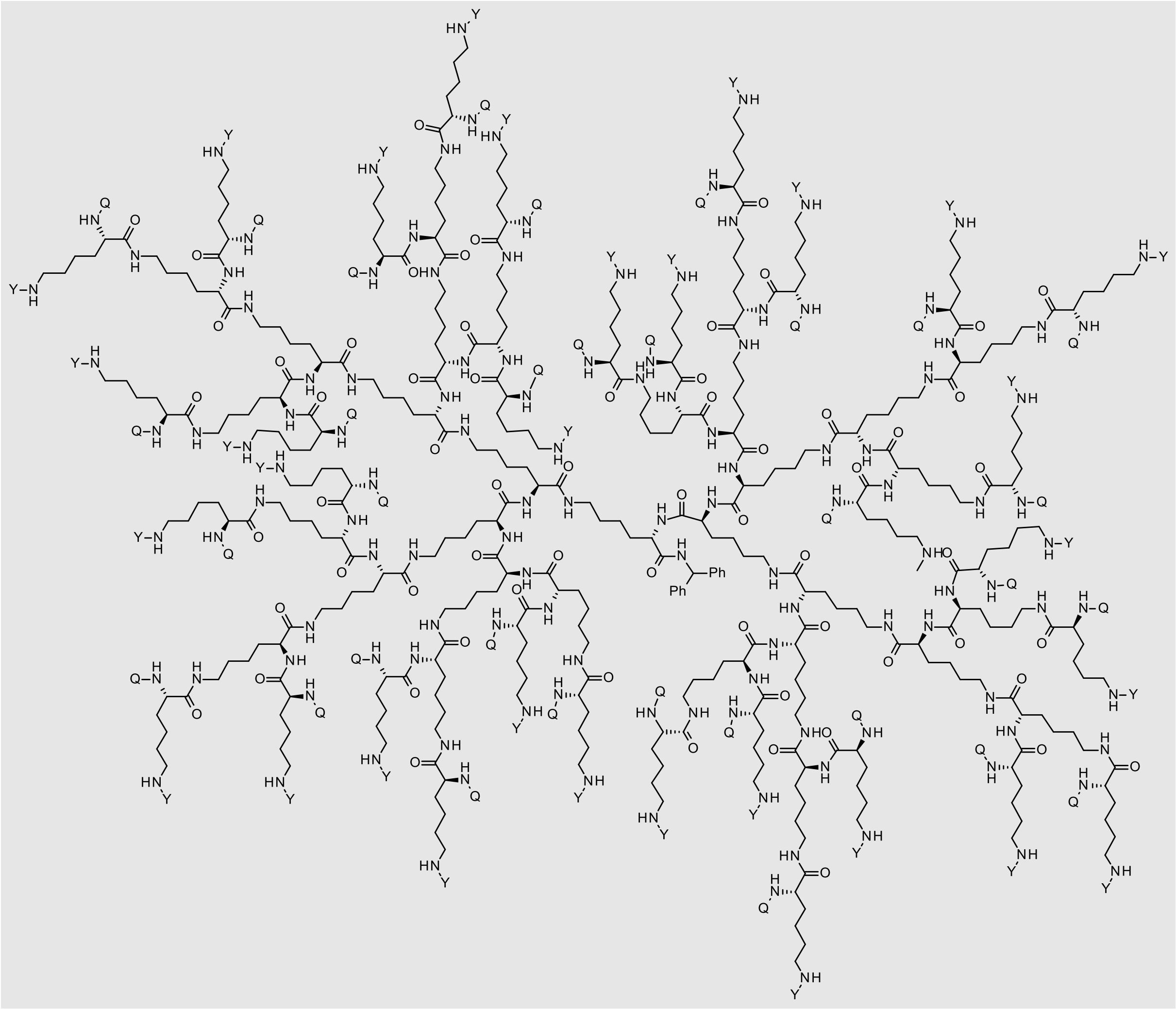

Supplementary Figure 3. **Structure of G5 PEGylated lysine dendrimer** showing conjugation points where PEG2100 at Y, and linker and drug at Q are attached.

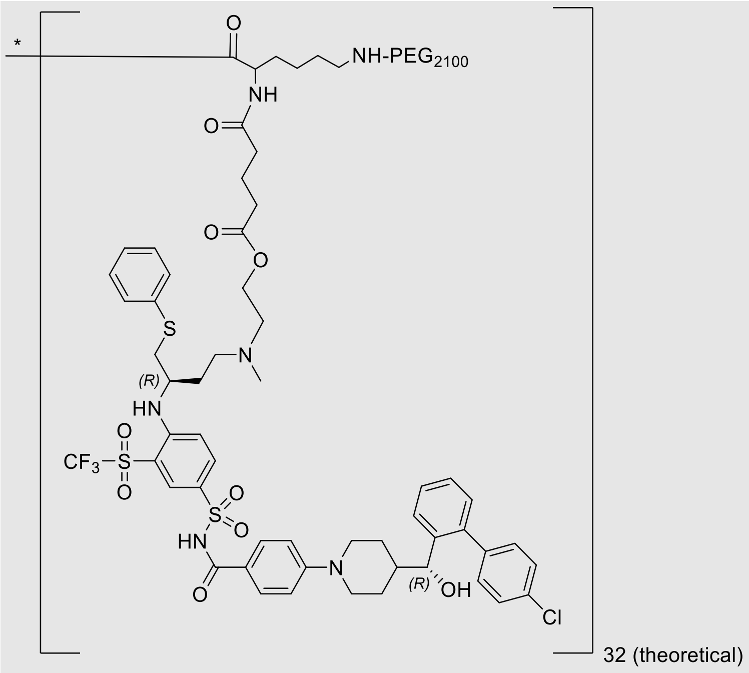

Supplementary Figure 4. Structure of glutarate linked AZD4230 dendrimer conjugate. SPL-8931.

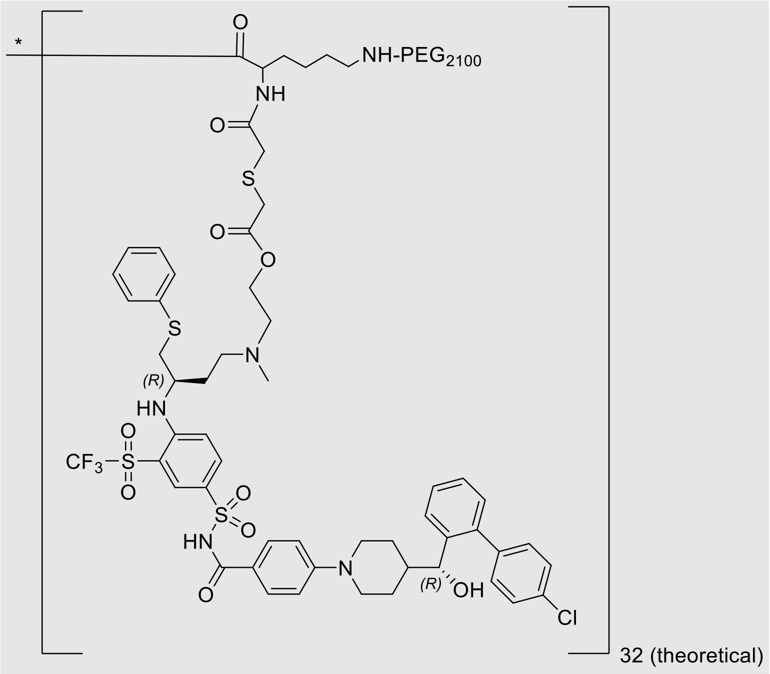

Supplementary Figure 5. Structure of thiodiglycolate linked AZD4230 dendrimer conjugate. SPL-8932.

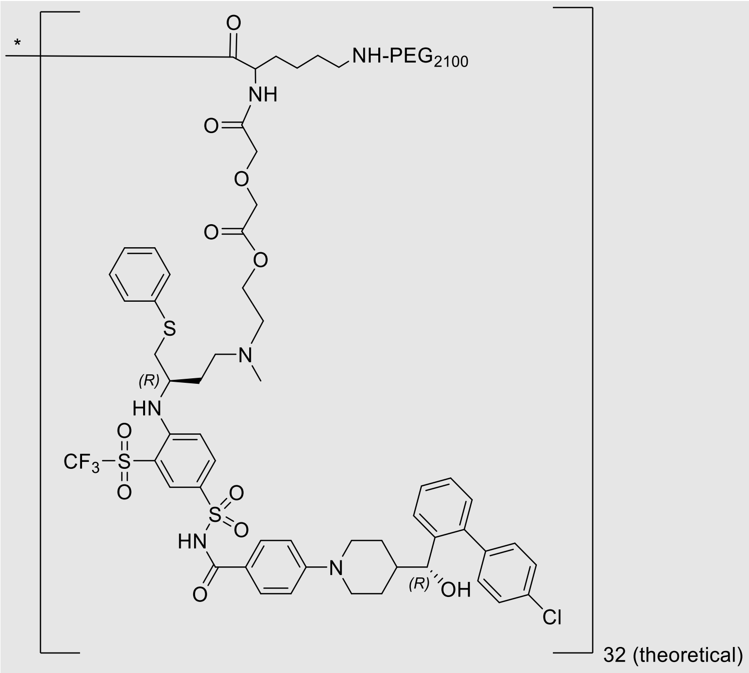

Supplementary Figure 6. Structure of diglycolate linked AZD4230 dendrimer conjugate, SPL-8933.

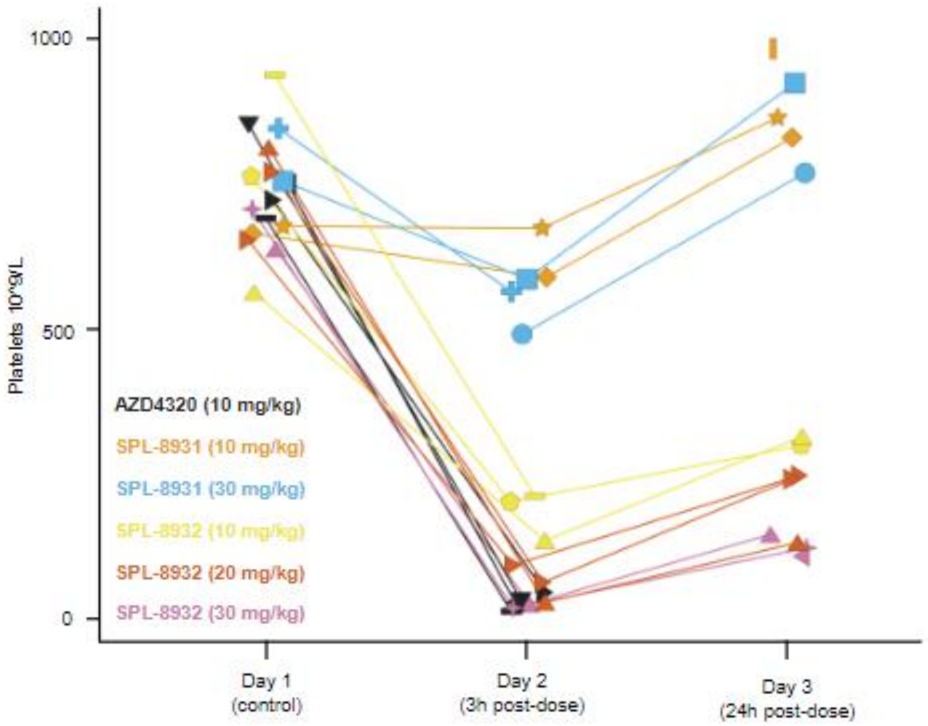

Supplementary Figure 7. Effect of AZD4320 and AZD4320-dendrimer conjugates on platelet count in rats. Data for dendrimers was collected from the same rats for which cardiovascular data were acquired (see Fig. 4a and b). Data for AZD4320 was collected from a non-cardiovascular study. Each symbol represent data from an individual rat. Colours representing each compound and dose are shown on the figure.

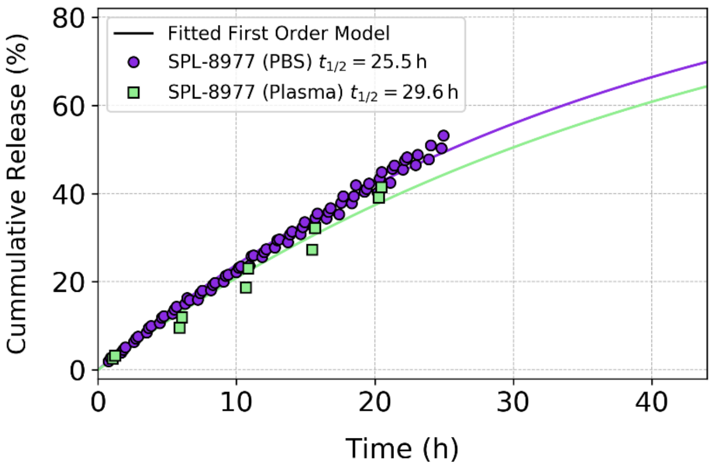

Supplementary Figure 8. Comparison and release rate in plasma and buffer. Cumulative in vitro release of AZD4320 measured over time from AZD4320-dendrimer conjugate SPL8977) in phosphate buffered saline (purple circles, n=3) and rat plasma (green squares, n=2) and fit to first order model at 37 °C (n=2)

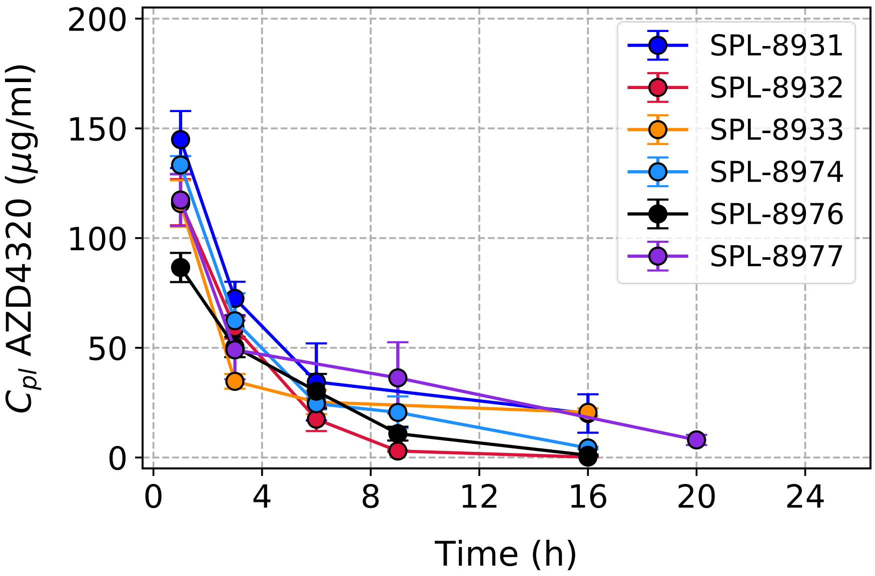

Supplementary Figure 9: Plasma pharmacokinetics of AZD4320 for all six dendrimers as total concentration of AZD4320 in plasma (conjugated + released), in C.B-17 SCID mouse at 10 mg/kg AZD4320 dose or equivalent (n=3 ± SD). The similar behaviour during the initial phase (up to 6 hours) shows that the release rate only plays a relatively small part in the decline of the total concentrations in plasma and suggests that RES uptake is a bigger factor, and this appears to be rather similar across all 6 dendrimers, justifying the use of a single k_{res} value across all 6 dendrimers.

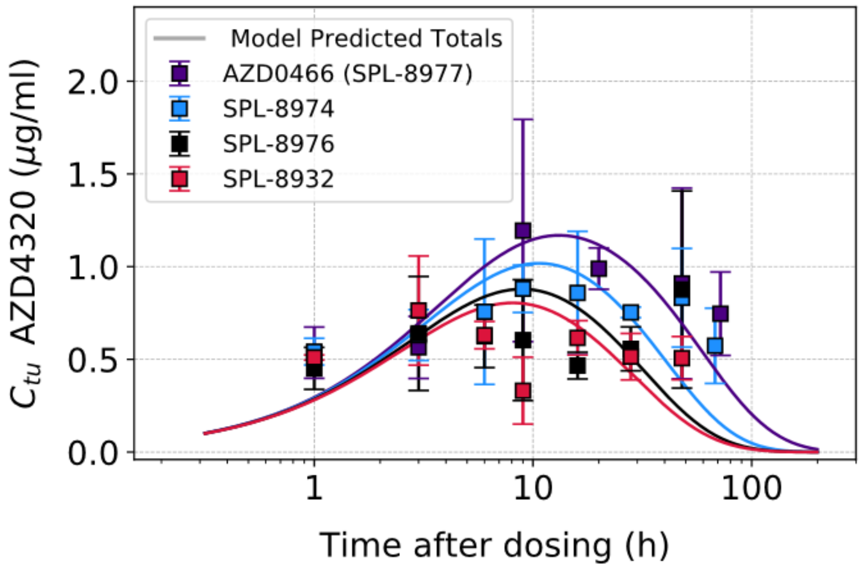

Supplementary Figure 10: Total (conjugated + released AZD4320) concentration in tumour tissue measured for AZD0466, SPL-8974, SPL-8976 and SPL-8932. The solid lines show the total concentrations predicted by the model (equation. Solid lines are pure predictions as no total levels were used in the model parameterisation. Dosed in C.B-17 SCID mouse at 10 mg/kg AZD4320 dose or equivalent (n=3 ± SD).

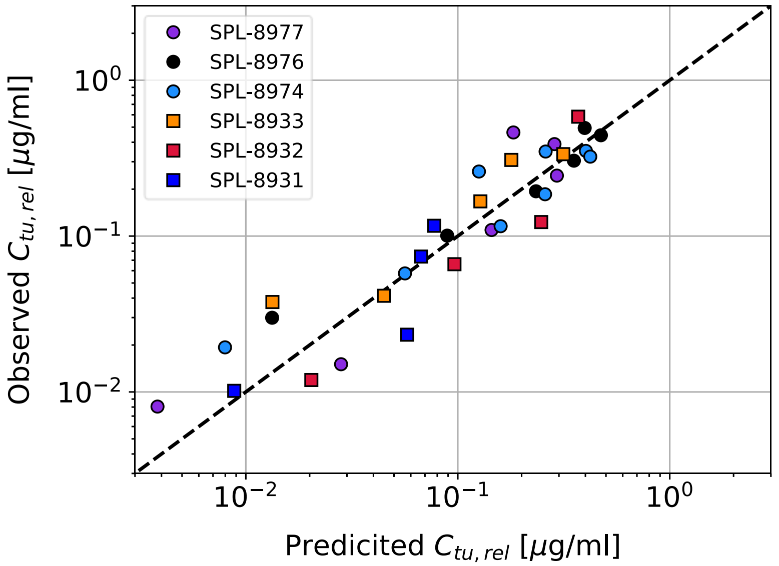

Supplementary Figure 11: Correlation plot comparing the model predictions for released AZD4320 at the tumour site verses the measured values at the same time point. The squares represent the measurements used to build the model and the circles the purely predicted values. The coefficient of determination, or R^2, for the full dataset is 0.65.

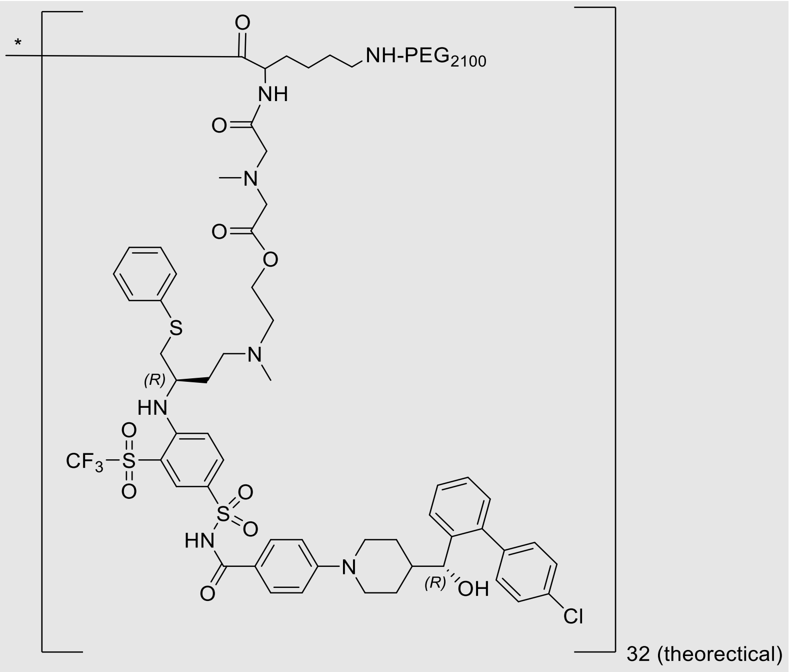

Supplementary Figure 12. Structure of N-methyl diacetate linked AZD4230 dendrimer conjugate. AZD0466 (formerly known as SPL-8977).

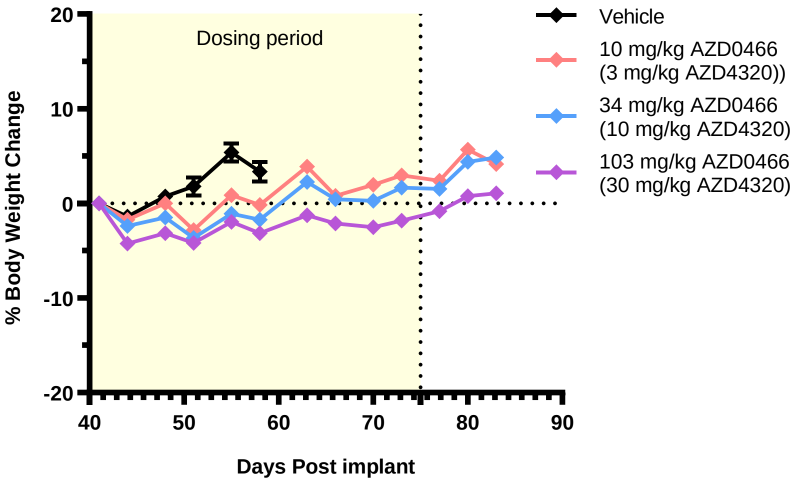

Supplementary Figure 13. Percent total body weight change in mouse RS4;11 xenograft model (n=6). Percent change in body weight of mice measured twice weekly after start of treatment with various doses of AZD0466.

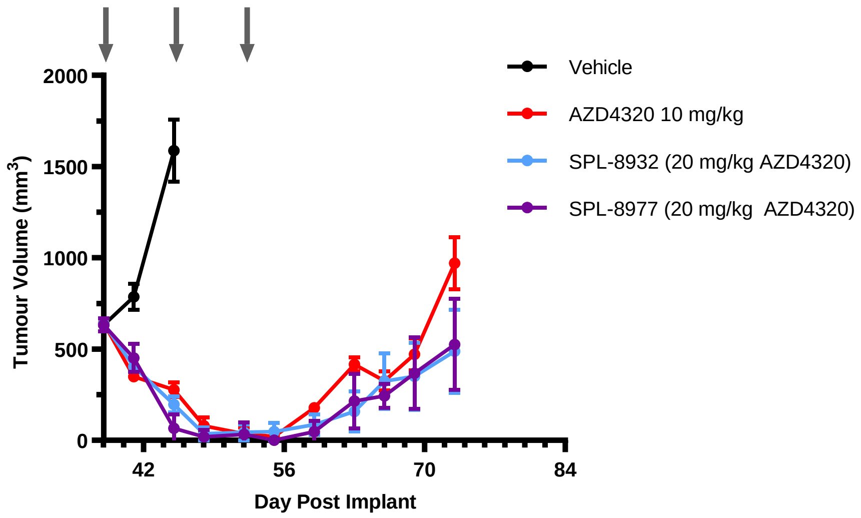

Supplementary Figure 14, Efficacy of AZD4320 and AZD4320-dendrimer conjugates in the mouse RS4;11 xenograft model. Tumour growth inhibition following dosing 20 mg/kg AZD4320 equivalent of SPL-8932 (blue) and SPL-8977 (AZD0466, purple) or 10 mg/kg of AZD4320 (red). Data in the graphs are represented as mean tumor volume ± SEM (n = 4 per group). Arrows indicate weekly dosing schedule.

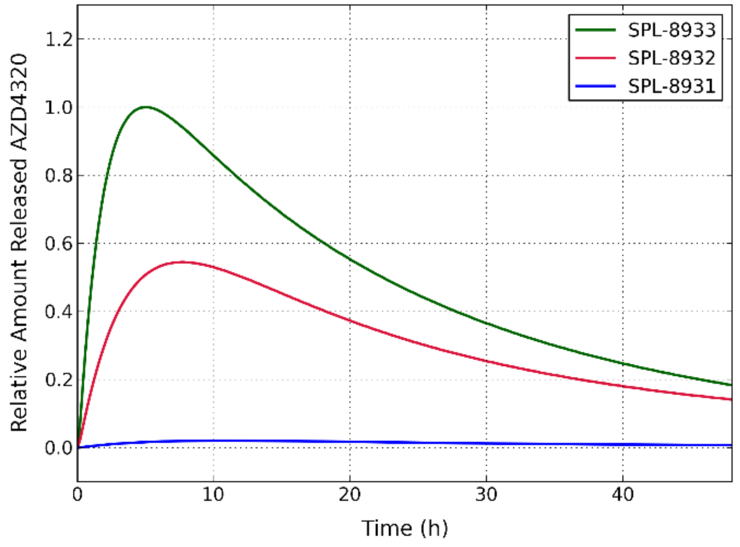

Supplementary Figure15: Predicted tumour profiles for AZD4320 from dosing drug-dendrimer conjugates if delivered via solely controlled release in plasma and not via extravasation in tumour

Supplementary Tables

Table S1. Characterisation of initial AZD4320-dendrimer conjugates.

SPL#	Dendrimer	MW (kDa)	Drug loading %	In vitro release $t_{1/2}$ (h)
SPL-8931	AZD4320-Glu-PEG_{2100}	104.5	28.2	201
SPL-8932	AZD4320-TDA-PEG_{2100}	106.0	28.6	4.4
SPL-8933	AZD4320-DGA-PEG_{2100}	105.6	28.8	1.7

Table S2. Summary of cardiovascular effects of AZD4320 and AZD4320-dendrimer conjugates in rats

	Control	AZD4320	SPL-8931		SPL-8932	
Release half-life (h)	NA	NA	217		5.4	
Dose (mg/kg)	NA	10	10	30	10	30
AZD4320 equivalent dose (mg/kg)	0	10	3	10	3	10
Tolerated?	Yes	6/8 tolerated	Yes	Yes	Yes	Yes
Platelets post-dose 10^9/l	709	30	630	546	182	22
QRS amplitude decrease	No	Yes	No	No	No	Yes
Blood pressure decrease	No	Yes	No	No	No	No

Table S3. Characterisation of AZD0466

AZD#	Dendrimer	MW (kDa)	Drug loading %	In vitro release $t_{1/2}$ (h)	Size (nm) Z_{ave}/PDI
AZD0466 (formerly SPL-8977)	AZD4320-MIDA-PEG_{2100}	109.0	23.5	25.5	10/0.08

Table S4. Cardiovascular effects of AZD4320 and AZD0466 in the dog.

	Infusion time (h)	Total dose (mg/kg)	AZD4320 equivalent dose (mg/kg)	Tolerated ?	Effects
AZD4320	0.5	0.2	0.2	Yes	No effect level
	0.5	0.85	0.85	Yes	↓QRS amplitude (49% decrease), ↓SBP, ↑HR, ↓dP/dt-, ↓PR interval, ↓QRS interval, ↓LVEDP
	0.5	1	1	No	↓QRS amplitude (75% decrease), poorly tolerated
	0.5	1.75	1.75	No	↓QRS amplitude (94% decrease), ↓SBP, ↑HR, ↓dP/dt-, ↓PR interval, ↓QRS interval, ↓LVEDP
	3	0.85	0.85	Yes	↓QRS amplitude (32% decrease), poorly tolerated
AZD0466	1	10	3	Yes	No effect level
	1	30	9	Yes	↓QRS amplitude (30% decrease)
	1	60	18	Yes	↓QRS amplitude (33% decrease), ↑DBP, ↑HR, ↓dP/dt+, ↓PR interval, ↓LVEDP

Table S5. LC-MS/MS parameters for analysis of AZD4320.

Mass spectrometer	Sciex API 5500		
Column	Waters XBridge C18 3.5 µm, 30 x 3mm at room temperature		
Solvent A	10 mM ammonium formate with 0.1% formic acid		
Solvent B	Acetonitrile with 0.1% formic acid		
Gradient	Time (min)	%A	%B
	0.2	90	10
	0.6	5	95
	0.8	5	95
	1.0	90	10
Flow	1.6 ml/min		
Run time	1.5 minutes		
