# Supplementary material for: Design and optimisation of dendrimer-conjugated Bcl-2/xL inhibitor, AZD0466, with improved therapeutic index for cancer therapy
Source: Commun Biol. 2021 Jan 25;4:112. doi: 10.1038/s42003-020-01631-8 (PMC7835349; doi:10.1038/s42003-020-01631-8)
Supplement: Supplementary file 3 — Description of Additional Supplementary Files [file 42003_2020_1631_MOESM3_ESM.pdf]

Description of Additional Supplementary Files

File name: Supplementary Data 1

Description: Raw data for the Figures.
