# Supplementary material for: Design and optimisation of dendrimer-conjugated Bcl-2/xL inhibitor, AZD0466, with improved therapeutic index for cancer therapy
Source: Commun Biol. 2021 Jan 25;4:112. doi: 10.1038/s42003-020-01631-8 (PMC7835349; doi:10.1038/s42003-020-01631-8)
Supplement: Supplementary file 5 — Reporting Summary [file 42003_2020_1631_MOESM5_ESM.pdf]

nature research

Corresponding author(s): Marianne Ashford

Last updated by author(s): Oct 19, 2020

Reporting Summary

Nature Research wishes to improve the reproducibility of the work that we publish. This form provides structure for consistency and transparency in reporting. For further information on Nature Research policies, see our Editorial Policies and the Editorial Policy Checklist.

Statistics

For all statistical analyses, confirm that the following items are present in the figure legend, table legend, main text, or Methods section.

n/a	Confirmed	
☐	☒	The exact sample size (n) for each experimental group/condition, given as a discrete number and unit of measurement
☐	☒	A statement on whether measurements were taken from distinct samples or whether the same sample was measured repeatedly
☐	☒	The statistical test(s) used AND whether they are one- or two-sided<br>*Only common tests should be described solely by name; describe more complex techniques in the Methods section.*
☒	☐	A description of all covariates tested
☐	☒	A description of any assumptions or corrections, such as tests of normality and adjustment for multiple comparisons
☐	☒	A full description of the statistical parameters including central tendency (e.g. means) or other basic estimates (e.g. regression coefficient) AND variation (e.g. standard deviation) or associated estimates of uncertainty (e.g. confidence intervals)
☐	☒	For null hypothesis testing, the test statistic (e.g. F, t, r) with confidence intervals, effect sizes, degrees of freedom and P value noted<br>*Give P values as exact values whenever suitable.*
☒	☐	For Bayesian analysis, information on the choice of priors and Markov chain Monte Carlo settings
☒	☐	For hierarchical and complex designs, identification of the appropriate level for tests and full reporting of outcomes
☒	☐	Estimates of effect sizes (e.g. Cohen's d, Pearson's r), indicating how they were calculated

Our web collection on statistics for biologists contains articles on many of the points above.

Software and code

Policy information about availability of computer code

Data collection	Dog telemetry signals were acquired and analysed using Ponemah software (St. Paul, Minnesota, USA). Rat telemrtry data acquisition used Dataquest Open A.R.T.™ with HEM software (Notocord Inc).
Data analysis	GraphPad PRISM for data analysis of the in vivo efficacy studies.<br>Berkeley Madonna, Version 8.3.14, University of California at Berkeley was used to generate PK parameters of AZD4320. Custom code was developed, for fitting model equations to experimental data used in the modelling, using Python 3.7.4 and the open source function library Scipy version 1.3.1. Full details of the Python code are available.

For manuscripts utilizing custom algorithms or software that are central to the research but not yet described in published literature, software must be made available to editors and reviewers. We strongly encourage code deposition in a community repository (e.g. GitHub). See the Nature Research guidelines for submitting code & software for further information.

Data

Policy information about availability of data

All manuscripts must include a data availability statement. This statement should provide the following information, where applicable:

- Accession codes, unique identifiers, or web links for publicly available datasets
- A list of figures that have associated raw data
- A description of any restrictions on data availability

The source data for Figs 2 - 8 and Supplementary Figs S2, S8, S9, S10, S11, S13, S14 & S15 are provided as a Source data file . Other data are available from the corresponding author upon reasonable request.

Field-specific reporting

Please select the one below that is the best fit for your research. If you are not sure, read the appropriate sections before making your selection.

- [x] Life sciences
- [ ] Behavioural & social sciences
- [ ] Ecological, evolutionary & environmental sciences

For a reference copy of the document with all sections, see nature.com/documents/nr-reporting-summary-flat.pdf

Life sciences study design

All studies must disclose on these points even when the disclosure is negative.

Sample size	Sample sizes were based on established experimental protocols and in keeping with IACUC guidelines on animal numbers
Data exclusions	No data was excluded
Replication	Animal studies were replicated in other instances with similar results
Randomization	Random allocation of animals based on tumor size to ensure even distribution and similar average tumor size between treatment arms prior to dosing for in vivo efficacy studies. IVDB Software tool was used to record tumor body weights and bioluminescence and for randomization.<br><br>For dog telemetry experiments, an ascending dose design was used. For some rat telemetry experiments, animals were randomized to receive either vehicle control or the test item. For other experiments, a cross-over study design was used, with an interval of 7 days between treatments.
Blinding	Blinding was not relevant as the investigator had to be aware of dosage and schedule which would identify the treatment.

Reporting for specific materials, systems and methods

We require information from authors about some types of materials, experimental systems and methods used in many studies. Here, indicate whether each material, system or method listed is relevant to your study. If you are not sure if a list item applies to your research, read the appropriate section before selecting a response.

Materials & experimental systems

n/a	Involved in the study	
	x	Antibodies
	x	Eukaryotic cell lines
x		Palaeontology and archaeology
	x	Animals and other organisms
x		Human research participants
x		Clinical data
x		Dual use research of concern

Methods

n/a	Involved in the study	
x		ChIP-seq
x		Flow cytometry
x		MRI-based neuroimaging

Antibodies

Antibodies used	Cleaved Caspase 3 Cell Signaling Technology (CST9661)<br>goat anti-rabbit Vector Labs (PK-6101)
Validation	Cleaved Caspase-3 (Asp175) Antibody detects endogenous levels of the large fragment (17/19 kDa) of activated caspase-3 resulting from cleavage adjacent to Asp175. This antibody does not recognize full length caspase-3 or other cleaved caspases. This antibody detects non-specific caspase substrates by western blot. Non-specific labeling may be observed by immunofluorescence in specific sub-types of healthy cells in fixed-frozen tissues (e.g. pancreatic alpha-cells). Nuclear background may be observed in rat and monkey samples.<br><br>Species Reactivity:<br>Human, Mouse, Rat, Monkey<br><br>Species predicted to react based on 100% sequence homology:<br>Bovine, Dog, Pig

Eukaryotic cell lines

Policy information about cell lines

Cell line source(s)	Ri-1 (DSMZ Cat# ACC-585, RRID:CVCL_1885); RS4;11 (ATCC Cat# CRL-1873, RRID:CVCL_0093); SUDHL-4 (DSMZ Cat# ACC-495, RRID:CVCL_0539); OCI-LY-10 (NIH/NCI RRID:CVCL_8795)
Authentication	STR was used for authentication
Mycoplasma contamination	Tested and passed
Commonly misidentified lines (See ICLAC register)	None

Animals and other organisms

Policy information about studies involving animals; ARRIVE guidelines recommended for reporting animal research

Laboratory animals	5-8 week old Female C.B.-17 SCID mice were purchased from Charles River Laboratories and used for PK and efficacy studies, Male Han Wistar rats were implanted with HD-S11 transmitters (Data Sciences International, St. Paul, MN USA) and used for tolerability studies. Dog telemetry studies were conducted in a pre-existing colony of beagle dogs previously surgically implanted with telemetry devices. Dogs were returned to the colony after each study for re-use. The test facility was Charles River Laboratories, Edinbugh UK/ Dogs were supplied by Envigo, Bicester, UK. The details are in the manuscript.
Wild animals	None
Field-collected samples	Not applicable
Ethics oversight	AAALAC, IACUC, Project license 40/3483 which has gone through the AstraZeneca Ethical Review Process

Note that full information on the approval of the study protocol must also be provided in the manuscript.
